# Hypothalamic–Pituitary–Thyroid Axis Crosstalk With the Hypothalamic–Pituitary–Gonadal Axis and Metabolic Regulation in the Eurasian Tree Sparrow During Mating and Non-mating Periods

**DOI:** 10.3389/fendo.2020.00303

**Published:** 2020-05-29

**Authors:** Ghulam Nabi, Yinchao Hao, Xuelu Liu, Yanfeng Sun, Yang Wang, Chuan Jiang, Juyong Li, Yuefeng Wu, Dongming Li

**Affiliations:** ^1^Key Laboratory of Animal Physiology, Biochemistry and Molecular Biology of Hebei Province, College of Life Sciences, Hebei Normal University, Shijiazhuang, China; ^2^Functional Laboratory, Experimental Center for Teaching, Hebei Medical University, Shijiazhuang, China; ^3^Ocean College, Hebei Agricultural University, Qinhuangdao, China

**Keywords:** hypothalamic–pituitary–thyroid axis, hypothalamic–pituitary–gonad axis, testis size, Eurasian tree sparrow, plasma metabolites, breeding sub-stages

## Abstract

Reproduction is an energetically costly phenomenon. Therefore, to optimize reproductive success, male birds invest enough energetic resources for maintaining well-developed testes. The hypothalamic–pituitary–thyroid (HPT) axis in birds can crosstalk with the hypothalamic–pituitary–gonadal (HPG) axis, thus orchestrating both the reproduction and metabolism. However, until now, how the free-living birds timely optimize both the energy metabolism and reproduction via HPT-axis is not understood. To uncover this physiological mechanism, we investigated the relationships among body mass, testis size, plasma hormones including thyroid-stimulating hormone (TSH), thyroxine (T_4_), triiodothyronine (T_3_), metabolites including glucose (Glu), triglyceride (TG), total cholesterol (TC), uric acid (UA), diencephalon mRNA expressions of type 2 (*Dio2*) and 3 (*Dio3*) iodothyronine deiodinase enzymes, thyrotropin-releasing hormone (*TRH*), thyroid-stimulating hormone (*TSH)*, gonadotropin-releasing hormone *I* (*GnRH-I)*, and gonadotropin-inhibitory hormone (*GnIH*) in a male Eurasian tree sparrow (ETS, *Passer montanus*). We found significantly larger testis size; elevated diencephalon *Dio2* and *TRH* mRNA expressions, plasma T_3_, and UA levels; and significantly lowered Glu, TG, and TC levels during mating relative to the non-mating stages in male ETSs. However, *Dio3, TSH, GnRH-I*, and *GnIH* mRNA expression did not vary with the stage. Furthermore, life-history stage dependent variation in plasma T_3_ had both direct effects on the available energy substrates and indirect effects on body mass and testis size, indicating a complex regulation of metabolic pathways through the HPT- and HPG-axes. The identified differences and relationships in mRNA expression, plasma T_3_ and metabolites, and testis size in male ETSs contribute to our understanding how free-living birds adjust their molecular, endocrinal, and biochemical features to orchestrate their reproductive physiology and metabolism for the maintenance of well-developed testes.

## Introduction

The hypothalamic–pituitary–thyroid (HPT) axis in both birds and other vertebrates appears to have similar functions in general (1). Thyroid hormones (THs) are considered as essential biomarkers of an animal energy expenditure (2). The activation of thyroid signaling modulates energy expenditure through both the central and peripheral pathways (3). In the mediobasal hypothalamus, triiodothyronine (T_3_), a bioactive form of THs, increased remarkably through conversion from thyroxine (T_4_) via down-regulation of the type 3 iodothyronine deiodinase (Dio3) and up-regulation of the type 2 iodothyronine deiodinase (Dio2) enzymes (4, 5). The increase of thyrotropin-releasing hormone (TRH) in the hypothalamus stimulates the production of thyroid-stimulating hormone (TSH) in the pars distalis (6), which further acts on the thyroid glands to secrete T_3_ and T_4_ THs (1, 6). Although, HPT-axis in birds regulates the basal metabolic rate (BMR) and several plasma metabolic parameters (7, 8). Still, the central molecular and cellular mechanisms regulating seasonality and metabolic adjustment to photoperiod are poorly understood (9).

The THs are believed to be a key metabolic regulator involving diverse metabolic pathways (10). In mammals, THs can coordinate energy needs through crosstalk with nuclear receptor signaling in various metabolic tissues or via a sympathetic pathway from the paraventricular nucleus to the liver (8, 11–13). Generally, higher levels of THs are associated with accelerated metabolism, weight loss, enhanced glucose (Glu) and protein metabolism, increased uptake of triglyceride (TG)-derived fatty acid in muscle and heart, and reduced plasma total cholesterol (TC) levels. In contrast, lower levels of THs shift the body to energy conservation mode (8). In birds, previous studies have demonstrated a similar effect of THs on metabolism, despite the underlying regulatory mechanism on metabolic signaling pathways remains unclear (1, 14, 15).

In the hypothalamus, TSH via T_3_ regulates the expression of RFamide-related peptides (RFRP) and Kisspeptin (KP) (16). The RFRP, KP, and gonadotropin-inhibitory hormone (GnIH) through interacting with gonadotropin-releasing hormone (GnRH) involving in the regulation of seasonal reproduction (17–20). In vertebrates, thyroid-axis during a photoperiodic cascade can regulate reproductive seasonality by activating the HPG-axis (photostimulation) and suppressing the HPG-axis (photorefractoriness) (4, 21, 22). The role of thyroid-axis in regulating reproductive seasonality is well understood in vertebrates (19, 23–25). However, the interaction between the hypothalamic TH-axis and HPG-axis to regulate the metabolism across the reproduction sub-stages in birds is not yet understood (20).

The energy demands of reproductive behavior, gametogenesis, and nestling are calorically expensive, and therefore, are regulated precisely in response to exogenous and endogenous cues (26). Plasma THs, ultimately, via thyroid receptors in the Sertoli cells of testes regulate photoperiodic gonadal growth and maturation and spermatogenesis (1, 19, 21). In male birds, the increase of TH concentrations is associated with the life-history stage with high energy demands and/or elevated BMR, such as when the testes are well developed during the mating period (3, 27). To maximize fitness, birds, therefore, invest enough nutritional and energetic resources into the development and maintenance of reproductive physiology and behavior (28). During the mating stage, male birds expressing an increase up to 480-fold in the testicular mass (29) suggests the higher energy demands for testicular growth and maintenance (28, 30). Meanwhile, the male bird has a pronounced swelling of the cloacal protuberance (31). In male temperate-zone monogamous birds, peak testosterone levels occurred coinciding with the time of female fertility (32). The increase in plasma T_3_ levels is fundamental to elevate BMR and provide extra energy requirements for maintaining higher levels of testosterone (7, 33). Still, the detailed mechanism of how the HPT-axis interact with the HPG-axis to regulate plasma metabolites and to orchestrate energetic cost at the mating stage in wild birds is unknown.

The Eurasian tree sparrow (ETS, *Passer montanus*) is a typical multiple-brooded species native to the Eurasian continent. The phenotypic traits in reproductive morphology and related physiology are associated with reproductive sub-stages (34–36). The ETSs had comparable mass-corrected BMR, but higher T_3_ levels during the early breeding relative to the late breeding stage (37). Our previous studies showed that testis size and plasma testosterone of male ETSs varied with breeding sub-stages, which are associated with body condition and corticosteroid-binding globulin (CBG; a proxy of energetic condition) (34, 35, 38). In addition, plasma metabolites including TC, TG, Glu, and uric acid (UA; a product of protein degradation) levels have been investigated and varied with external environmental conditions (39).

In the present study, our objectives were (1) to investigate the relationships among body mass, the height (CPH), and the width (CPW) of the cloacal protuberance; testis size; diencephalon *GnRH-I* and *GnIH* mRNA expression; plasma metabolites (Glu, TG, TC, and UA); and HPT-axis activity (plasma T_3_, T_4_, and TSH and diencephalon *TRH, TSH, Dio2*, and *Dio3* mRNA expression) in male ETSs during breeding period; (2) to determine the differences in all these measured variables between mating (egg-laying stage of females) and non-mating stages; and (3) to evaluate the causations of interactions between HPT and HPG-axes through modulating plasma metabolites in male ETSs during mating and non-mating stages. To date, given that little information is available on the correlations between body mass or testis size and HPT-axis activity and plasma metabolites it is therefore challenging to investigate the causations among these variables in free-living animals. To solve this problem, we developed a structural equation model [SEM; a powerful tool for biologists to test causation and correlation experiments; (40)] comprised of a latent variable (available energy substrates) which can explain correlation among variable and complete path diagrams of causation. We predicted that (1) male sparrows contribute more energy for spermatogenesis during the mating stage, so they would express lower energetic condition reflected by decreased plasma TG and TC levels and increase UA levels relative to the non-mating stages; (2) enhanced HPT-axis activity reflected by increased *Dio2* mRNA and plasma THs would be necessary to compensate for such elevated energetic condition during mating relative to non-mating stages; and (3) larger testis size of male sparrows during breeding season could be positively correlated with plasma metabolites, and T_3_ and diencephalon *GnRH-I* and *Dio2* mRNA.

## Materials and Methods

### Animal Collections

A total of 24 adult male ETSs during breeding period were caught opportunistically using a Japanese mist nets in 2014, between April 23 and June 24 (day length, the minimum and maximum temperature of free-living conditions are shown in Figure S1), at the campus of Hebei Normal University (37°59.88′N, 114°31.18′E, elevation: 72 m), Shijiazhuang, Hebei Province, China. To reduce the effects of circadian rhythm, we caught the birds between 0600 and 1,000 h. According to the timing of reproductive behavior and anatomy of this species in our previous studies (34, 38, 41), we further divided the breeding period into the non-mating (the nest building stage, April 23, *n* = 6; the early nestling stage, June 4–5; *n* = 6) and mating stages (the early egg-laying stage, May 19; *n* = 6; the late egg-laying stage, June 24; *n* = 6). In male ETSs at sampling site, these breeding sub-stages are relatively synchronized according to our field observation of reproductive behaviors and reproductive anatomy (e.g., females have follicular yolk deposition and males show significantly increased testes size during egg-laying periods). Male sparrows were identified by the absence of a brood patch as it is a female-specific feature.

### Morphological Measurement and Tissue Sampling

Within 10 min after capture in the field, each bird was weighed to ±0.01 g. The CPW and CPH were measured to ±0.01 mm using a vernier caliper. After morphological measurements, each animal was individually housed in a cage (40 × 30 × 30 cm), provided with foxtail millet (*Setaria italica*) and water *ad libitum*, and transferred to a laboratory at Hebei Normal University within 2 h of capture.

When the birds were transferred to the laboratory, approximately 150 μL of blood was collected into heparinized micro-hematocrit capillary tubes from the alar vein following venipuncture with a 26-gauge needle. Blood samples were stored on ice for 1–2 h until centrifugation at 855 × *g* for 10 min. Plasma was aspirated, split into four fractions, and stored at −80°C until analysis. Immediately following blood sampling, birds were euthanized with phenobarbitone (7.5 μL g^−1^ body mass). The diencephalon was immediately excised and frozen in liquid nitrogen and stored at −80°C until RNA extraction. The short axis and long axis of the left and right testis size were measured to ±0.01 mm. The testis size of each individual was used and calculated according to the formula of 4/3π*a*^2^*b*, where *a* is half of the short axis of the testis and *b* is half of the long axis. The maximum testis size (either from the left or right side) was used in the following analysis.

After the birds were caught for about 2 h, blood samples were collected for measuring plasma hormones and metabolites and brains for measuring several genes expression of HPT- and HPG-axes. The blood and brain samples were collected at the same time, which enabled us to compare the differences and investigate their potential interactions. All protocols were approved by the Ethics and Animal Welfare Committee (no. 2013-6) and by the Institutional Animal Care and Use Committee (HEBTU2013-7) of Hebei Normal University, China, and were conducted under the auspices of scientific collecting permits issued by the Departments of Wildlife Conservation (Forestry Bureau) of Hebei Provinces, China.

### Gene Cloning of *Dio2, Dio3, TRH, TSH, GnRH-I*, and *GnIH* for the ETS

Total RNA was extracted from diencephalons using the TRIZOL reagent (Invitrogen, Carlsbad, CA, USA) and then reverse transcribed to cDNA using the SuperScript III RT kit (Invitrogen) according to the manufacturer's instructions. After the reaction, the cDNA was diluted 10 times for polymerase chain reaction (PCR). The fragments of the coding region of the *Dio2, Dio3, TRH, TSH, GnRH-I*, and *GnIH* were amplified by PCR using primers in Table S1. The PCR primers (Table S1) were designed in the conserved regions of corresponding gene sequences deposited in GeneBank of other passerines such as zebra finch (*Taeniopygia suttata*) and white-throated sparrow (*Zonotrichia albicollis*) and obtained the open reading frames for *Dio2, Dio3, TRH, TSH*, and *GnRH-I* in ETSs (the nucleotide sequences for each gene were deposited in NCBI, Table S1) by aligning the corresponding sequences using Primer Premier 5.0 (PREMIER Biosoft International, Palo Alto, CA, USA). All PCR amplifications were performed in a 50-μL reaction mixture containing 2.0 μL cDNA (200 ng/μL), 5.0 μL mixed dNTPs (2.5 mM each), 10.0 μL 5 × Fast Pfu Buffer and 2.0 μL forward and reverse primers (10 mM), respectively, 1 μL Trans Start FastPfu DNA polymerase (2.5 U/μL), and 28.0 μL sterile water.

### qPCR of *Dio2, Dio3, TRH, TSH, GnRH-I*, and *GnIH* for the ETS

The qPCR reactions were set up using the TransStrat Top Green qPCR SuperMix (Quanshijin, Beijing, China). PCR was performed in a 200-μL Eppendorf tube containing 0.5 μL of each primer, 12.5 μL 2 × TransStarTM Top Green qPCR SuperMix (contains Taq DNA polymerase, reaction buffer, dNTP mix, Passive Reference Dye II, and 10 mM MgCl_2_), and 4 μL cDNA in a total volume of 25 μL. The amplification proceeds in a two-step cycle (Predegeneration at 94°C for 30 s, denaturation at 94°C for 5 s, annealing and extension at 60°C for 30 s, and data collection at 72°C for 45 s) for 40 cycles. The primers used in qPCR reactions for *TRH, TSH, Dio2, Dio3, GnRH-I, GnIH*, and β*-actin* (reference gene) were shown in Table S2.

To determine the mRNA expression of *Dio2, Dio3, TRH, TSH, GnRH-I*, and *GnIH*, CT values of these genes in each sample were calculated and the transcript levels were calculated by the 2-ΔΔCT method. Endogenous relative expression was normalized to β-actin and the fold-change from the control group CT value.

### Assays of Plasma Hormones and Metabolites

The content of plasma T_3_, T_4_, and TSH was determined using enzyme immunoassay kits (Haling, Shanghai, China) according to the manufacturer's instruction, respectively (Cat No. for T_3_: HLE94014; T4: HLE94016, TSH: HLE94018). Briefly, 20 μL of plasma was diluted five times before assays. Standard curves with five dilutions ranging from 0.05 to 6 ng/mL for T_3_, 0.5 to 60 ng/mL for T_4_, and 2 to 24 pg/mL for TSH were obtained from each sample. All samples were run in triplicates. Intra- and inter-assay variations were 2.05 and 4.52% for T_3_, 1.26 and 6.73% for T_4_, 1.41 and 5.49% for TSH, respectively. Assay sensitivity was 0.110 ng/mL for T_3_, 2.93 ng/mL for T_4_, and 0.205 pg/mL for TSH.

The content of plasma Glu, TG, TC, and UA was measured using an automatic biochemical analyzer with commercially available kits (Mindray BS-180, Mindray Corp., Shenzhen, China). Plasma samples (20 μL) were diluted 40 times with dH_2_O and assayed with commercially available kits for Glu, TG, TC, and UA, respectively (Mindray Corp., Shenzhen, China). All samples were run in duplicates. Intra- and inter-assay variation were 2.84 and 7.81% for Glu, 7.3 and 9.5% for TG, 6.7 and 9.1% for TC, and 5.9 and 10.8% for UA, respectively. Assay sensitivity was 0.3 mmol/L for Glu, 0.03 mmol/L for TG, 0.04 mmol/L for TC, and 14.2 μmol/L for UA.

### Statistical Analysis

Spearman correlations were used to investigate relationships among body mass, CPW, CPH, testis size, plasma T_3_, T_4_, TSH, Glu, TG, TC, UA, and mRNA expression of *Dio2, Dio3, TRH, TSH, GnRH-I*, and *GnIH* in the brain during the breeding season. We used independent *t*-tests to evaluate the differences in body mass, CPW, CPH, testis size, plasma T_3_, T_4_, TSH, and mRNA expression of *Dio2, Dio3, TRH, TSH, GnRH-I*, and *GnIH* in the diencephalon between the mating and non-mating stages, respectively. If the values did not meet the assumption of homogeneity of variances, Welch's *t*-test was used. In consideration that 17 parameters for Spearman correlations and 17 *t*-tests were run in this study, *p*-values were then corrected for false positives by the Benjamini-Hochberg method for robust results.

To identify the relationships between body mass or testis size and plasma THs and metabolites, we constructed a SEM in the *lavaan* package (42) of Program R v.3.4.2, including all combinations of those important variables as identified by AIC scores, and selected the best model with the Chi-square, comparative fit index (CFI), root mean square error of approximation (RMSEA). A well-fitted model should have a *p*-value > 0.05 in the Chi-square test, an RMSEA of <0.1, and a CFI closer to 1 (>0.9). In the SEM, we defined the available energy substrates as the latent variable comprising plasma Glu, TG, TC, and UA. We further employed a discrete variable by dividing the whole breeding stage into two sub-stage, i.e., mating vs. non-mating season, to evaluate the effect of reproductive status. The relationships between available energy substrates and plasma metabolites (Glu, TG, TC, and UA) were represented by factor loading; the effects of available energy substrates, plasma T_3_, and mating stage on body mass or testis size were represented by regression coefficients; and all path coefficients used standardized estimates. All tests were performed using the Program R v.3.4.2. The *p*-values or adjusted *p*-values < 0.05 were considered significant. All data are presented as the mean ± 95% confidence interval.

## Results

### Correlations Among All Measured Variables During the Breeding Season

**Figure 1:**
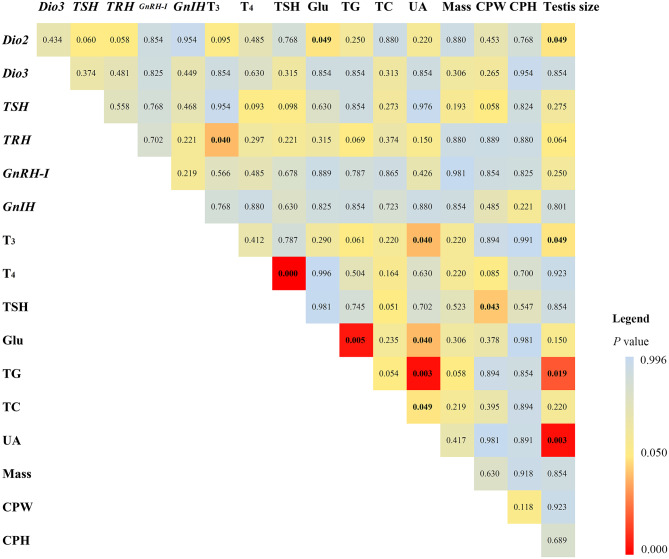
Statistical results of Spearman correlation (adjusted *p*-values are shown) in mRNA expression of type 2 iodothyronine deiodinase (*Dio2*), type 3 iodothyronine deiodinase (*Dio3*), thyrotropin-releasing hormone (*TRH*), thyroid-stimulating hormone (*TSH*), gonadotropin-releasing hormone *I* (*GnRH-I*), gonadotropin-inhibitory hormone (*GnIH*) in the diencephalon, plasma triiodothyronine (T_3_), thyroxine (T_4_), TSH, glucose (Glu), triglyceride (TG), total cholesterol (TC), uric acid (UA), body mass, the width (CPW) and height (CPH) of the cloacal protuberance, and testis volume in breeding male Eurasian tree sparrows (*Passer montanus*). The legend shows the distinguishable *p*-values in different colors.

Among the correlation results of 17 measured variables (Figure 1), plasma T_3_ levels positively correlated with diencephalon *TRH* mRNA expression (ρ = 0.641), plasma UA levels (ρ = 0.638), and testis size (ρ = 0. 639), respectively. Plasma TSH levels positively correlated with plasma T_4_ levels (ρ = 0.807), but negatively with CPW (ρ = −0.582). Diencephalon *Dio2* mRNA expression positively correlated with testis size (ρ = 0.621), but negatively with plasma Glu levels (ρ = −0.581). Plasma Glu and TG levels positively correlated (ρ = 0.721). Plasma UA levels negatively correlated with plasma Glu (ρ = −0.604), TG (ρ = −0.568), and TC levels (ρ = −0.568), respectively. Testis size negatively correlated with plasma TG levels (ρ = −0.723) but positively with UA levels (ρ = 0.782).

### Comparisons of All Measured Variables Between Mating and Non-mating Stages

**Figure 2:**
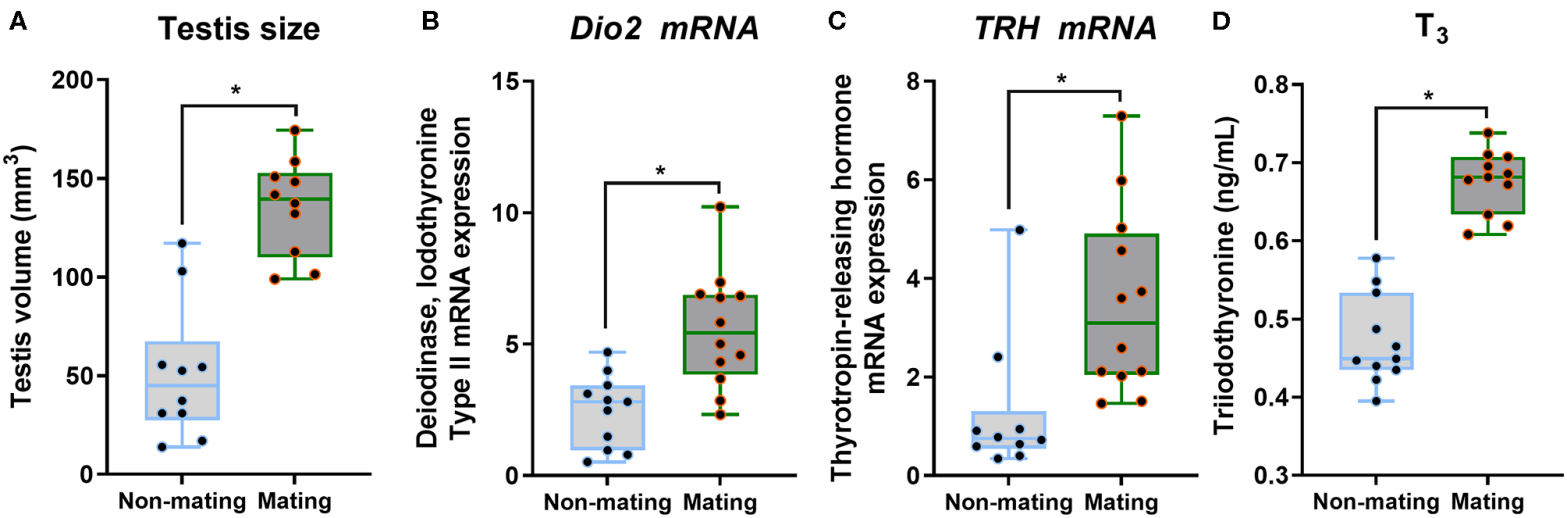
Comparisons of testis size **(A)**, the type 2 iodothyronine deiodinase (*Dio2*; **B**) mRNA, thyrotropin-releasing hormone (*TRH*; **C**) mRNA in the diencephalon, and plasma triiodothyronine (T_3_; **D**) between the non-mating and mating stages in male Eurasian tree sparrows (*Passer montanus*). Asterisk represents a significant difference between groups (*p* < 0.05).

**Figure 3:**
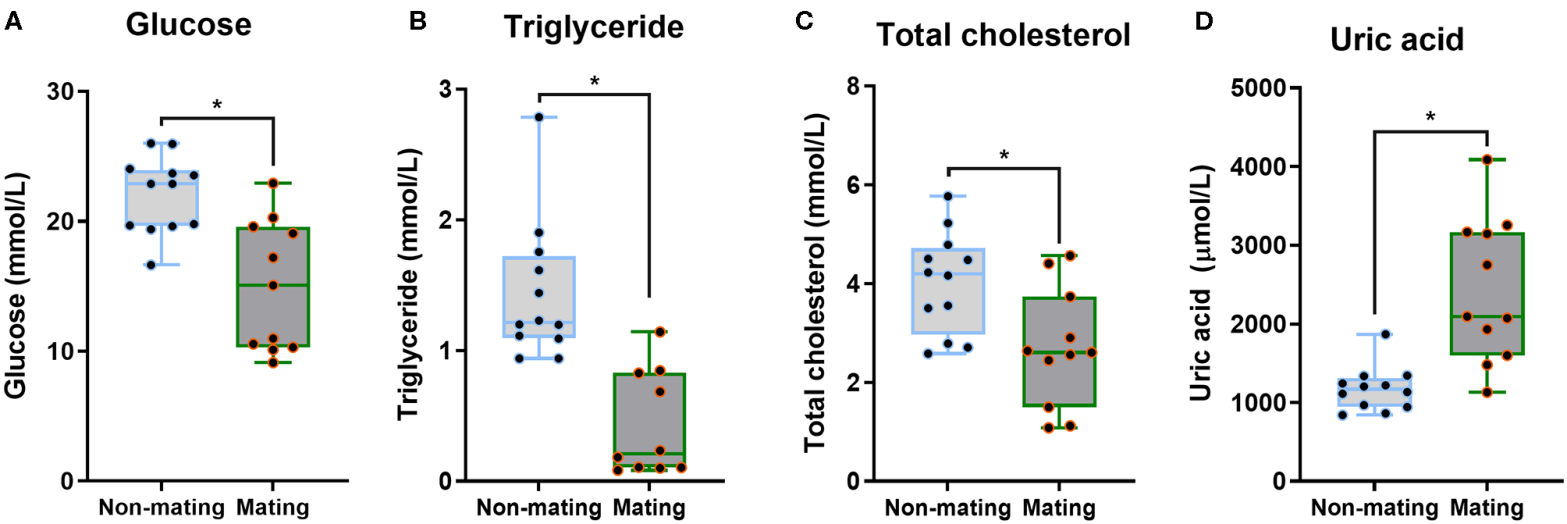
Comparisons of plasma glucose **(A)**, triglyceride **(B)**, total cholesterol **(C)**, and uric acid **(D)** between the non-mating and mating stages in male Eurasian tree sparrows (*Passer montanus*). Asterisk represents a significant difference between groups (*p* < 0.05).

The body mass, CPW, and CPH of male ETSs did not change; however, testis sizes were significantly larger during the mating compared to the non-mating stages (Table 1; Figure 2A). In the diencephalon, *Dio3, TSH, GnRH-I*, and *GnIH* mRNA expression did not vary with the stage. However, the *Dio2* and *TRH* mRNA expression during the mating stage were significantly higher relative to the non-mating stage (Table 1; Figures 2B,C). Plasma T_4_ and TSH levels did not change; however, T_3_ levels increased significantly during the mating stage relative to the non-mating stage (Table 1; Figure 2D). Plasma Glu, TG, and TC levels decreased while UA levels increased remarkably in the mating stage relative to the non-mating stage (Table 1; Figure 3).

**Table 1:** Statistical results of independent *t*-test in body mass, testis size, mRNA expression of type 2 iodothyronine deiodinase (*Dio 2*), type 3 iodothyronine deiodinase (*Dio 3*), thyrotropin-releasing hormone (*TRH*), thyroid-stimulating hormone (*TSH*), gonadotropin-releasing hormone (*GnRH-I*), and gonadotropin–inhibitory hormone (GnIH) in the diencephalon, plasma triiodothyronine (T_3_), thyroxine (T_4_), TSH, glucose (Glu), triglyceride (TG), total cholesterol (TC), and uric acid (UA) in male Eurasian tree sparrows (*Passer montanus*) between the non-mating and mating stages.

**Type of variable**	**Variable**	***t*-value**	***df***	***p*-value**	**Ajusted *p*-value**
Morphology	Body mass	−1.888	21.688	0.073	0.138
	CPW	0.544	18.863	0.593	0.672
	CPH	0.219	14.824	0.830	0.830
	Testis size	6.304	16.347	**9.50E-06**	**8.08E-05**
mRNA expression in the diencephalon	*Dio 2*	4.053	18.528	**0.001**	**0.003**
	*Dio 3*	0.726	17.570	0.478	0.580
	*TRH*	3.156	19.857	**0.005**	**0.012**
	*TSH*	0.990	15.117	0.338	0.442
	*GnRH-I*	1.557	11.223	0.147	0.250
	*GnIH*	0.371	22	0.714	0.759
Plasma thyroid hormones	T_3_	9.586	17.905	**1.79E-08**	**3.043E-07**
	T_4_	1.245	21.768	0.226	0.320
	TSH	1.294	17.611	0.212	0.320
Plasma metabolites	Glu	−4.049	15.841	**0.001**	**0.003**
	TG	−5.073	19.867	**5.92E-05**	**3.35E-04**
	TC	−2.881	19.808	**0.009**	**0.020**
	UA	4.374	11.693	**0.001**	**0.003**

*Significant effects (P < 0.05) are shown in bold*.

### Relationships Among Plasma T_3_, Metabolites, Body Mass, and Testis Size in Mating and Non-mating Stages

**Figure 4:**
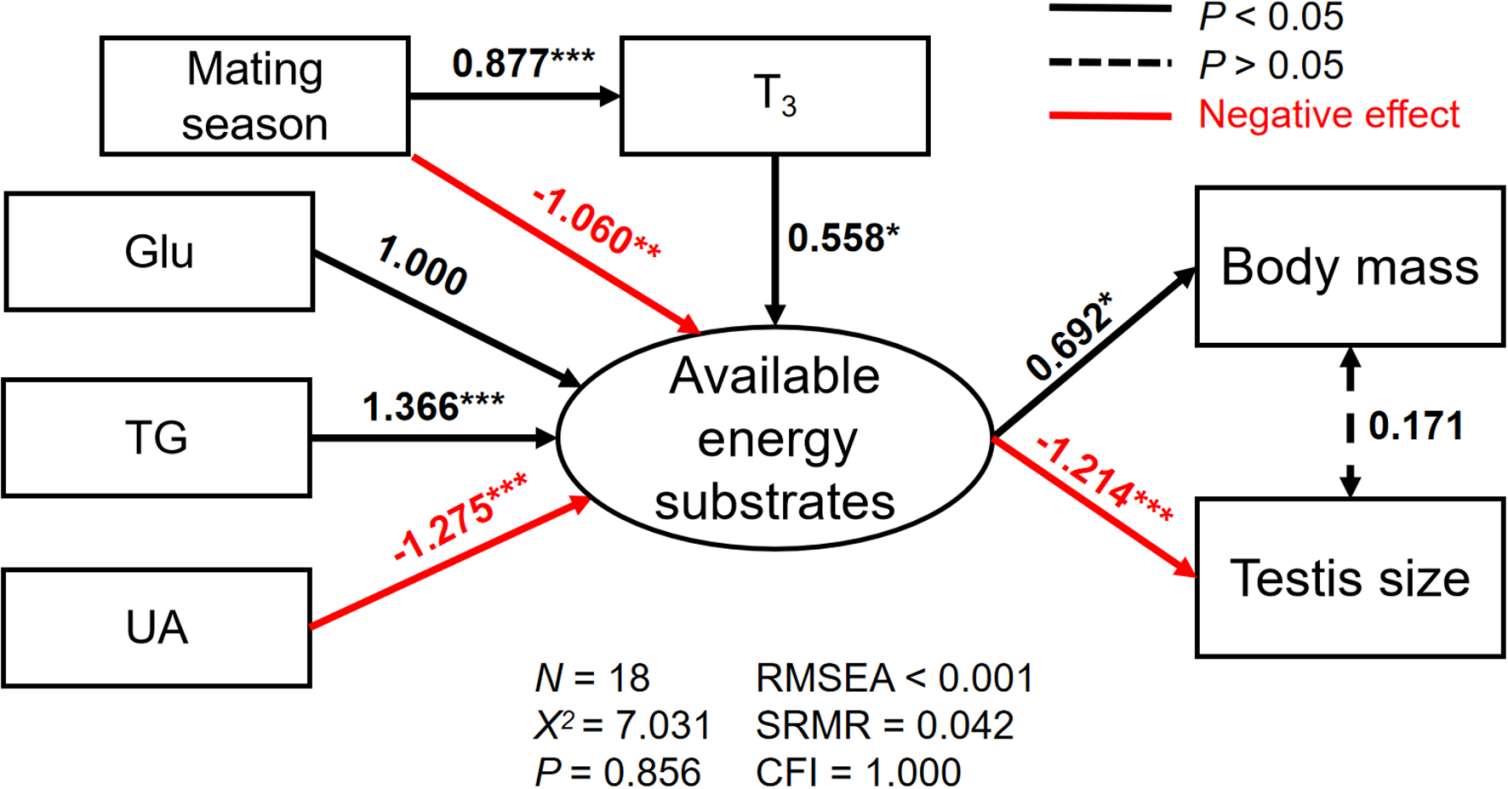
The relationships among plasma triiodothyronine (T_3_), glucose (Glu), triglyceride (TG), and uric acid (UA), and their direct effects on the latent variable (the available energy substrates), and indirect effects on either body mass (a) or testis size (b) for male Eurasian tree sparrows (*Passer montanus*) during breeding in the best-fit structural equation model. RMSEA, root mean square error of approximation; SRMR, standard root mean square residual; CFI, comparative fit index. *p < 0.05; **p < 0.01; and ***p < 0.001.

The best SEM for explaining the variations in body mass and testis size consisting five well-fitted variables including reproductive status, plasma T_3_, Glu, TG, and UA (χ^2^ = 7.031, df = 12, *p* = 0.856; RMSEA < 0.001, SRMR = 0.042, CFI = 1.000; Figure 4). We found the available energy substrates were significantly determined by plasma Glu, TG, and UA but not TC levels. Specifically, plasma Glu and TG were positively related while plasma UA was negatively related to the available energy substrates (Figure 4). Furthermore, an increase in plasma T_3_ during the mating season was directly associated with the available energy substrates that are necessary for a positive effect on body mass. Similarly, larger testes size results from more consumption of the available energy substrates (Figure 4; Tables S3–S5).

## Discussion

### Interactions of HPT-axis Activity, Plasma T_3_, Metabolites, Body Mass, and Testis Size in the Breeding Period

Both the central and peripheral pathways of the HPT-axis modulate energy expenditure (3) and, therefore, varied with life-history stages of an individual to maximize fitness (3, 27, 28). Among the measured variables in the HPT-axis, we detected significantly positive relationships between plasma T_3_ and diencephalon TRH mRNA expression, and between plasma T_4_ and plasma TSH levels. Like our study, such relationships among diencephalon TRH, plasma TSH, and plasma THs (T_3_ and T_4_) have been confirmed in several vertebrate species (43–45).

Generally, the levels of circulating plasma metabolites can help us to understand the instantaneous metabolic condition of an individual (46). We detected a negative relationship between diencephalon *Dio2* mRNA expression and plasma Glu levels and a positive relationship between plasma T_3_ and UA levels. Our results confirmed the notion of accelerated metabolism through mobilizing plasma Glu and protein catabolism induced by higher plasma T_3_ or brain *Dio2* mRNA expression (8, 10). Furthermore, we found that male ETSs exhibit a positive relationship between plasma Glu and TG and negative relationships between plasma UA and Glu, TG, and TC levels. Among vertebrates, the general mechanism is that when Glu is exhausted, the individuals turn to adipose tissues as their principal metabolic fuel and then the protein catabolism and conversion of proteins into lipids (47, 48). Therefore, lower plasma Glu levels can result from enhanced metabolic rate of catabolism, which may be compensated by lipid mobilization (49). In addition, enhanced protein catabolism reflected by the increase of UA in birds (49–53) may occur when glucose and lipids are in shortage. Our results suggest complex, one after another, relationships among carbohydrate, lipid, and protein metabolisms during the breeding period in male ETSs.

The enlargement in testicular size in birds is believed to be associated with the higher mating frequency and more sperm production for maintaining higher testosterone levels secreted from Leydig cells in testes (54–56). We detected testis size positively correlated with diencephalon *Dio2* mRNA expression, plasma T_3_ and UA levels, and negatively with plasma TG levels, whereas CPW negatively correlated with plasma TSH levels. Unexpectedly, testis size neither positively correlates with diencephalon *GnRH-I* mRNA expression, nor negatively with diencephalon *GnIH* mRNA expression, which is in line with the findings of Moore et al. [61)]. During spring (photostimulation), medio-basal hypothalamic TSHβ and GnRH-I secretion increases which further increases gonadal maturation (57, 58). However, during photorefractoriness, GnRH-I synthesis ceases and the gonads regress (58). In contrast to GnRH-I, GnIH in song sparrows (*Melospiza melodia*) and house sparrows (*Passer domesticus*) suggests an inhibitory role in regulating seasonal breeding (59); however, in wild Australian zebra finches, no difference was observed in the synthesis of GnIH mRNA across the reproductive stages, indicating differences in their reproductive strategy (60). TH is believed to be a key metabolic regulator involving diverse metabolic pathways such as protein, carbohydrate, and lipid metabolism (10). The HPT-axis via thyroid receptors acts on the Sertoli and Leydig cells of testes, regulate photoperiodic gonadal growth and maturation and steroidogenesis and spermatogenesis (61).

### Differences Among All Measured Variables Between Mating and Non-mating Stages

In this study, male ETSs did not express significant differences in the levels of diencephalon *GnRH-I* and *GnIH* mRNA expression between mating and non-mating stages. However, male ETSs exhibited significantly larger testicular size during the mating relative to non-mating stages. In male ETSs, higher testosterone levels at the onset of breeding season (34, 41) could be related to increasing the intensity and frequency of sexual display and reproductive aggression (62–65). The stage-dependent testis enlargement and mass-related costs of carrying them around could be a proxy of enhanced metabolic expense (55). Although, a detailed study including daily activities and BMR is lacking in this free-living species; however, our results indicate higher energy investment in the testicular maintenance at the mating period.

To cope with the higher energetic costs required for testicular maintenance (29, 66–68), the BMR is elevated by activating the HPT-axis (4, 30, 33). The higher concentration of T_3_ in birds has been reported to elevate BMR and provide extra energy during the breeding season (33). We here observed up-regulation of the *TRH* and *Dio2* mRNA expression in the diencephalon and higher plasma T_3_ levels in male ETSs in the mating relative to the non-mating stages. In addition, we also detected remarkably lower plasma Glu, TG, TC, but higher UA levels during the mating compared with the non-mating stages. The decrease in plasma TG may reflect the individuals inhibit the synthesis and promote the breakdown of TG, thus providing non-esterified fatty acids for fatty acid oxidation and glycerol for continued gluconeogenesis (69, 70) during the mating stage. In addition, plasma TC is generally obtained from the diet or hepatic production (71) and may preferentially be directed toward steroidogenesis and spermatogenesis (72, 73). Our results support the notion of enhancing protein catabolism to replenish amino acid substrates for gluconeogenesis when plasma Glu is exhausted (74). Therefore, the increase in activity of the HPT-axis in relation to the decrease in plasma glycolipid metabolites indicates that ETSs invest more energy during the mating stage through activating HPT-axis to modulate energy expenditure.

### Relationships Among Plasma T_3_, Metabolites, Body Mass, and Testis Size in Mating and Non-mating Stages

Our SEM not only found that plasma Glu, TG, and UA levels can reflect the available energy substrates, but also revealed the causality of plasma T_3_, mating season, the available energy substrates, body mass, and testis size (Figure 4). Our results revealed that a life-history stage dependent variation in plasma T_3_ had both direct effects on the available energy substrates and indirect effects on body mass and testis size, indicating a complex regulation of metabolic pathways through HPT- and HPG-axes. In particular, the decrease in plasma Glu and TG levels and the increase in UA levels resulting from increased metabolic capacity during the mating season would contribute to an enlarged testis size. Similarly, eutherian mammals also exhibited a positive relationship between testes size and mass-specific metabolic rate (75). Although, we did not measure mass-specific metabolic rate; however, an increase in the THs during breeding enhances BMR to provide extra energy for highly energy-demanding behavior (68). Therefore, our results indicate male sparrows during mating season can further up-regulate the HPT-axis activity to modulate the available energy substrates for energy requirements for reproductive activities such as mating behavior.

### Limitations

In this study, we assumed that all the birds have experienced 2-h acute stress of capture and restraint in the cages without food and water deprivation. Although in our previous studies several physiological parameters of the breeding Eurasian tree sparrows exhibited irregular changes in response to acute stress, some of them could return to their normal states within the first hour of capture stress (34, 38, 39, 41). Given that some physiological parameters could have changed during capture, handling, and restraint stress, therefore, further investigations are warranted to consider the effects of stress-induced physiological alternations on both HPT- and HPG-axes during the breeding period.

## Conclusion

To our knowledge, this study takes the first to evaluate the interaction between the HPT- and the HPG-axes in male free-living animals during the breeding season. In male ETSs, the increase in testis size, plasma T_3_ and UA levels and decrease in Glu, TG, and TC during the mating stage relative to non-mating stage indicate a complex regulation in HPT- and HPG-axes through dynamic changes of metabolic pathways. The identified differences in *Dio2* mRNA expression in the diencephalon, plasma T_3_, metabolites, and testis size and no differences in *Dio3, TSH, GnRH-I*, and *GnIH* mRNA expression between mating and non-mating stages in male ETSs contribute to our understanding how free-living birds adjust their molecular, endocrine, and biochemical features to orchestrate their reproductive physiology for the maintenance of well-developed testes in free-living birds. Further studies are needed to investigate the detailed mechanism in the regulation of metabolic capacity between the interactions of the HPT- and the HPG-axes at the hypothalamic, pituitary, and gonadal levels during different breeding sub-stages.

## Data Availability Statement

All datasets generated for this study are included in the article/Supplementary Material.

## Ethics Statement

All protocols were approved by the Ethics and Animal Welfare Committee (no. 2013-6) and by the Institutional Animal Care and Use Committee (HEBTU2013-7) of Hebei Normal University, China, and were conducted under the auspices of scientific collecting permits issued by the Departments of Wildlife Conservation (Forestry Bureau) of Hebei Provinces, China.

## Author Contributions

DL and YWu conceived the study. GN analyzed the data and drafted the article with the help of YWa, CJ, and JL. YH, XL, and YS conducted the fieldwork and lab assays. DL and YWu helped in writing the manuscript and critically reviewed the article. All authors read and approved the final manuscript.

## Conflict of Interest

The authors declare that the research was conducted in the absence of any commercial or financial relationships that could be construed as a potential conflict of interest.
