# Supplementary material for: Hypothalamic–Pituitary–Thyroid Axis Crosstalk With the Hypothalamic–Pituitary–Gonadal Axis and Metabolic Regulation in the Eurasian Tree Sparrow During Mating and Non-mating Periods
Source: Front Endocrinol (Lausanne). 2020 May 29;11:303. doi: 10.3389/fendo.2020.00303 (PMC7272604; doi:10.3389/fendo.2020.00303)
Supplement: Supplementary file 1 [file Data_Sheet_1.pdf]

Supplementary Material

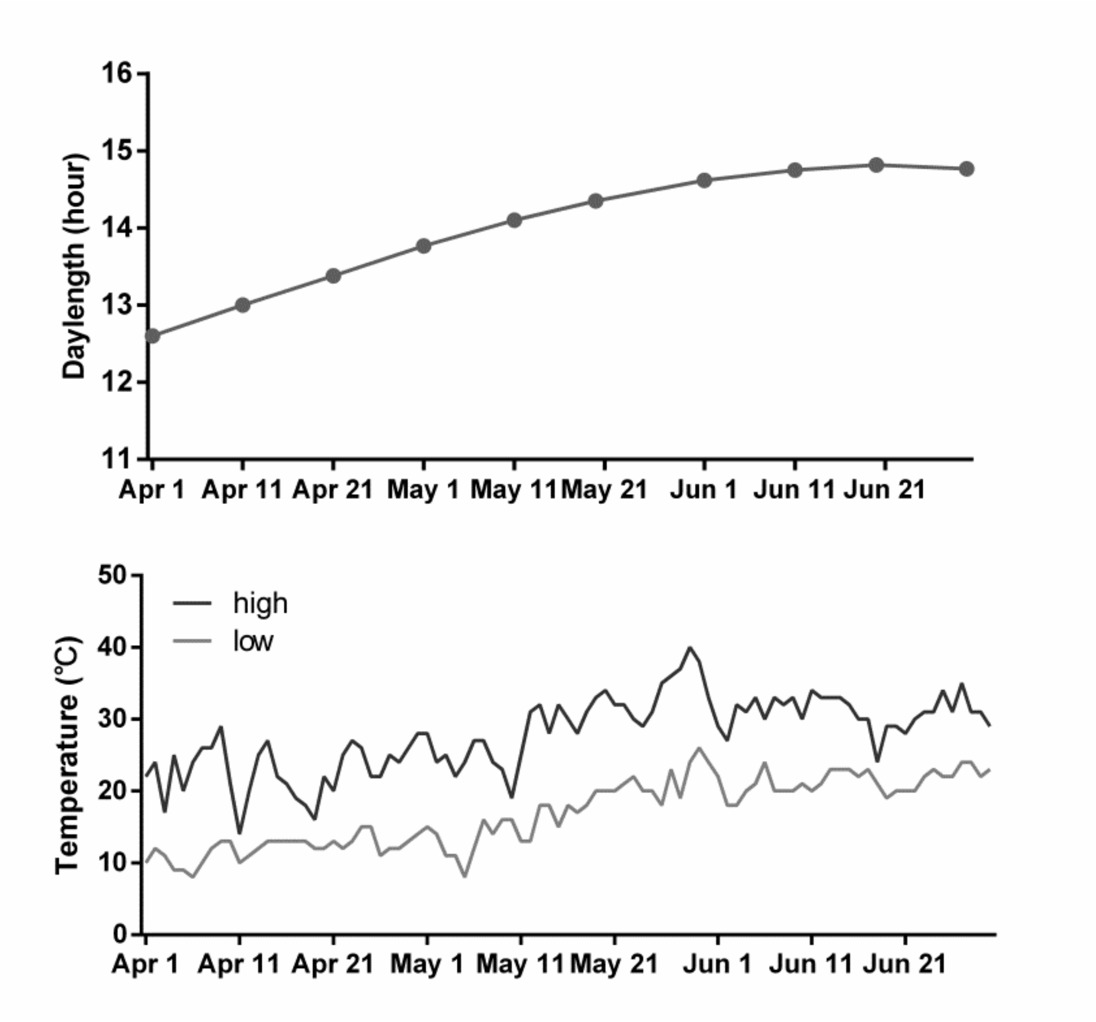

Fig.S1 The day length, minimum and maximum temperature of field environments (Shijiazhuang, Hebei Province, China) where the free-living Eurasian tree sparrow (*Passer montanus*) were sampled from between April 1 to June 30, 2014.

Table S1 The sense and antisense PCR primers used to clone *Dio2*, *Dio3*, *TRH*, *TSH*, *GnRH -I*, and *GnIH* cDNA (the coding regions of each gene were shown in Appendix A) for the Eurasian tree sparrow (*Passer montanus*) (The).

Gene	Primer	Sequence (5' to 3')
TRH	Sense 1	TGCCASTGCTACTCCTYTG
	Antisense 1	CTGYCTCTTCTGAATGTCTCCA
	Sense 2	CACTGCTACTCCTTTGCTTGACCTC
	Antisense 2	GCTTTTCTTCTACCCTGTCTCTGCT
	Sense 3	CCAGCTGCCACTGCTACTCC
	Antisense 3	TGCCTCTTCTGAATGCCTCC
	Sense 4	CTCATTCTTCAGTCTGTCCT
	Antisense 4	TTCTCATTCCTCTGTTTCAC
	Sense 5	TCAAGGTCATCTTCCATCTCCC
	Antisense 5	ACAATGCCATCCATCCAGCT
TSH	Sense 1	ATGAGTCCCTTCTTTGTGAT
	Antisense 1	GTAGTTTGTCCTGACCCTCT
	Sense 2	CCATCCCCTACTACTCCTAC
	Antisense 2	TGAGCAGAGCAGTACATTTC
Dio2	Sense 1	GGAAGATGGGTTTGCTAAGTG
	Antisense1	TGCATCACCTTAAAGGGACA
Dio3	Sense1	AGCACAAGCCGCTRCGAT
	Antisense1	TTGCCAGGTCCTTTTTACA
GnRH	Sense	GCAGTTACTCCACAACCTCTCTCAG
	Antisense	AACCTCACTCTGGCTCTGTGGCTCC
GnIH	Sense	ACAGATTCTGAGCTCTCTAAGTAAG
	Antisense	ACCAATAGGACATAAGTAGCACA
**ß-actin**	Sense	TGCTATGTCGCCCTGGATTTC
	Antisense	GGATACCACAGGACTCCATACCC

Table S2 The sense and antisense primers of *TRH*, *TSH*, *Dio2*, *Dio3*, *GnRH*, *GnIH*, and β-actin for quantitative polymerase chain reactions (qPCR) in diencephalon of male Eurasian tree sparrow (*Passer montanus*).

Gene	Primer	Sequence (5' to 3')
TRH	Sense	CTGCTACAACGGCTTTCCAA
	Antisense	GCCTCTTCTGAATGCCTCCA
TSH	Sense	CTCACCACACCATCCCCTAC
	Antisense	CTGACCCTCTCACGGACACA
Dio2	Sense	CGCCTGGAATCTCTCCCTCT
	Antisense	ACTCGCTCAAATGAAACCCC
Dio3	Sense	CAGAGGACCAGAGGGCTACA
	Antisense	CACACGGCTTTCAGCGACT
GnRH	Sense	GGGACCGTTCCAGGTGATTC
	Antisense	CTTCTGCCTTGTTCCTCCGC
GnIH	Sense	AAGACTGGGGAACAAAAGACATC
	Antisense	CCTGTGTGATACCTTTGGAGCAT
**β-actin**	Sense	CACAGATCATGTTTGAGACCTTCA
	Antisense	GATGGGCACAGTGTGGGTAAC

Table S3. Regression results of plasma triiodothyronine (T_3), the latent variable (metabolism), mating season in the structural equation model explaining variation in body mass and testis size for Eurasian tree sparrows (*Passer montanus*).

Regression	Variable	Estimate	Se	*z*	*p* value
**T_3**	Mating season	0.877	0.097	9.055	**<0.001**
**Metabolism**	T_3	0.558	0.262	2.130	**0.033**
	Mating season	-1.060	0.340	-3.119	**0.002**
**Body mass**	Metabolism	0.692	0.333	2.079	**0.038**
**Testis size**	Metabolism	-1.214	0.371	-3.276	**0.001**

Table S4. Statistical effects of latent variable (metabolism) including Glu, TG, UA in the structural equation model explaining variation in body mass and testis size for Eurasian tree sparrows (*Passer montanus*).

Latent variable	Variable	Estimate	Se	*z*	*p* value
**Metabolism**	Glu	1.000			
	TG	1.366	0.383	3.568	**<0.001**
	UA	-1.275	0.384	-3.316	**0.001**

Table S5. Covariances between body mass and testis size in the structural equation model explaining variation in body mass and testis size for Eurasian tree sparrows (*Passer montanus*).

Covariances		Estimate	Se	*z*	*p* value
**Body mass**	**Testis size**	0.171	0.118	1.449	0.147

Appendix A Coding region of TRH, TSH, Dio2, Dio3, GnRH-I, GnIH of Eurasian tree sparrow

LOCUS Seq1 762 bp mRNA linear VRT 10-NOV-2019

DEFINITION Thyrotropin releasing hormone (TRH) mRNA, complete cds.

ACCESSION Seq1

VERSION

KEYWORDS .

SOURCE Passer montanus (Eurasian tree sparrow)

ORGANISM Passer montanus

Eukaryota; Metazoa; Chordata; Craniata; Vertebrata; Euteleostomi;

Archelosauria; Archosauria; Dinosauria; Saurischia; Theropoda;

Coelurosauria; Aves; Neognathae; Passeriformes; Passeroidea;

Passeridae; Passer.

REFERENCE 1 (bases 1 to 762)

AUTHORS hao,y.

TITLE Direct Submission

JOURNAL Submitted (10-NOV-2019) Key Laboratory of Animal Physiology,

Biochemistry and Molecular Biology of Hebei Province, College of

Life Sciences, Hebei Normal University, Shijiazhuang 050024,

China., universities, No. 20, South Second Ring Road, Shijiazhuang,

Hebei Province, shijiazhuang, hebei province 050024, China

COMMENT Bankit Comment: ALT EMAIL:haoyinchao001@126.com

Bankit Comment: TOTAL # OF SEQS:1

##Assembly-Data-START##

Sequencing Technology :: Sanger dideoxy sequencing

##Assembly-Data-END##

FEATURES Location/Qualifiers

source 1..762

/organism="Passer montanus"

/mol_type="mRNA"

/db_xref="taxon:9160"

/country="China: Shijiazhuang,Hebei,province"

/altitude="176 m."

/collected_by="Yinchao Hao"

gene 1..762

/gene="Thyrotropin releasing hormone (TRH)"

CDS 1..762

/gene="Thyrotropin releasing hormone (TRH)"

/note="The hormone is a kind of pituitary hormone secreted by the hypothalamus, which promotes the secretion of thyroid-stimulating hormone from the pituitary and further controls the synthesis of thyroid hormone."

/codon_start=1

/product="Thyrotropin releasing hormone (TRH)"

/translation="MPSIQLPLLLLCLTSCGVCFNGGHLLPEESENMGKSPLDHILQR

SESLILQSVLKKAEEINKDSNAPVLQRLSKRQHPGEKYLSNLEKRQHPGKRDVEEETS

YGGIQKRQHPGKREMEDDLDVYLGLKRQQPSSRKSLLDQFAYSPRAQLTYMNELSKTE

HPGRRYPMYKHQRPSKRGWNYEVDVYDEKRQHPGKRHWNSDSSDDTGPCNFQDSLTCH

KGSLLLDLIDVSRDRVEEKRQHPGKRSAWESETEG"

BASE COUNT   249 a   164 c   189 g   160 t

ORIGIN

        1 atgccatcca tccagctgcc actgctactc ctttgcttga cctcgtgtgg tgtttgcttc

       61 aacgggggac atctccttcc agaggagagt gagaacatgg gaaaaagtcc cctagatcac

      121 atccttcaga gatctgaaag cctcattctt cagtctgtcc tcaagaaagc tgaagagatt

      181 aataaagact caaatgcccc tgtgctacaa cggctttcca aaagacaaca ccctggggaa

      241 aagtacctaa gtaacctgga aaagagacag catcctggaa aaagagatgt tgaggaagag

      301 acatcttatg gaggcattca gaagaggcag catcccggaa aaagggagat ggaagatgac

      361 cttgatgtct atctggggtt gaaaaggcaa cagccttcca gcagaaagtc actgttggat

      421 cagtttgctt acagtcctag ggcacagcta acttacatga acgagttatc caaaacagaa

      481 catccaggca gaagatatcc aatgtacaag caccagcgtc ctagcaaaag aggctggaat

      541 tatgaggtag atgtatatga tgagaaacgc cagcatcctg ggaaaaggca ctggaattct

      601 gacagctcag atgacacagg tccctgcaat tttcaggact cactcacttg tcacaaaggc

      661 agcttgttgc ttgatttaat agatgttagc agagacaggg tagaagaaaa gcgtcagcac

      721 ccaggaaaga gatcagcatg ggaaagtgaa acagagggat ga

//

LOCUS   Seq1   408 bp   mRNA   linear   VRT 11-NOV-2019

DEFINITION thyroid stimulating hormone beta (TSHB) mRNA, complete cds.

ACCESSION   Seq1

VERSION

KEYWORDS   .

SOURCE   Passer montanus (Eurasian tree sparrow)

  ORGANISM Passer montanus

            Eukaryota; Metazoa; Chordata; Craniata; Vertebrata; Euteleostomi;

            Archelosauria; Archosauria; Dinosauria; Saurischia; Theropoda;

            Coelurosauria; Aves; Neognathae; Passeriformes; Passeroidea;

Passeridae; Passer.

REFERENCE 1 (bases 1 to 408)

AUTHORS hao,y.

TITLE Direct Submission

JOURNAL Submitted (11-NOV-2019) Key Laboratory of Animal Physiology,

Biochemistry and Molecular Biology of Hebei Province, College of

Life Sciences, Hebei Normal University, Shijiazhuang 050024,

China., universities, No. 20, South Second Ring Road, Shijiazhuang,

Hebei Province, shijiazhuang, hebei province 050024, China

COMMENT Bankit Comment: ALT EMAIL:haoyinchao001@126.com

Bankit Comment: TOTAL # OF SEQS:1

##Assembly-Data-START##

Sequencing Technology :: Sanger dideoxy sequencing

##Assembly-Data-END##

FEATURES Location/Qualifiers

source 1..408

/organism="Passer montanus"

/mol_type="mRNA"

/db_xref="taxon:9160"

/country="China: Shijiazhuang,Hebei,province"

/altitude="176 m."

/collected_by="Yinchao Hao"

gene 1..408

/gene="TSHB"

/note="thyroid stimulating hormone subunit beta"

CDS 1..408

/gene="TSHB"

/note="Thyroid stimulating hormone is a hormone secreted

by the pituitary gland to promote the growth and function

of the thyroid gland."

/codon_start=1

/product="thyroid stimulating hormone beta (TSHB)"

/translation="MSPFFVMSLLFGLIFGQTASLCAPAEYTIHVEKRECAYCLAINT

TICAGFCMTRDSNGKKKLLLKSALSQNVCTYKDMLYRTALIPGCPLHTIPYYSYPVAL

SCKCGKCNTDYSDCVRERVRTNYCTKPQKLCNL"

BASE COUNT 95 a 120 c 99 g 94 t

ORIGIN

1 atgagtccct tctttgtgat gtctctcctc tttggcctga tttttggaca aacagcatca

61 ctctgtgctc ctgccgagta cacaattcac gtggagaagc gggaatgtgc ctattgcctg

121 gccatcaaca ccaccatctg cgctggattc tgcatgactc gggacagcaa tggcaagaag

181 aagctgctcc tgaagagtgc tctgtcccag aacgtgtgca cgtacaagga catgctgtac

241 cggacagcgc tgatcccggg ctgccctctc cacaccatcc cctactactc ctaccccgtg

301 gctctgagct gcaagtgtgg caagtgcaac actgactaca gtgactgtgt ccgtgagagg

361 gtcaggacaa actactgcac taagccacag aagctctgta acctgtaa

//

LOCUS Seq1 840 bp mRNA linear VRT 11-NOV-2019

DEFINITION iodothyronine deiodinase 2 (DIO2) mRNA, complete cds.

ACCESSION Seq1

VERSION

KEYWORDS .

SOURCE Passer montanus (Eurasian tree sparrow)

ORGANISM Passer montanus

Eukaryota; Metazoa; Chordata; Craniata; Vertebrata; Euteleostomi;

Archelosauria; Archosauria; Dinosauria; Saurischia; Theropoda;

Coelurosauria; Aves; Neognathae; Passeriformes; Passeroidea;

Passeridae; Passer.

REFERENCE 1 (bases 1 to 840)

AUTHORS hao,y.

TITLE Direct Submission

JOURNAL Submitted (11-NOV-2019) Key Laboratory of Animal Physiology,

Biochemistry and Molecular Biology of Hebei Province, College of

Life Sciences, Hebei Normal University, Shijiazhuang 050024,

China., universities, No. 20, South Second Ring Road, Shijiazhuang,

Hebei Province, shijiazhuang, hebei province 050024, China

COMMENT Bankit Comment: ALT EMAIL:haoyinchao001@126.com

Bankit Comment: TOTAL # OF SEQS:1

##Assembly-Data-START##

Sequencing Technology :: Sanger dideoxy sequencing

##Assembly-Data-END##

FEATURES Location/Qualifiers

source 1..840

/organism="Passer montanus"

/mol_type="mRNA"

/db_xref="taxon:9160"

/country="China: Shijiazhuang,Hebei,province"

/altitude="176 m."

/collected_by="Yinchao Hao"

gene 1..840

/gene="DIO2"

/note="iodothyronine deiodinase 2 (DIO2)"

CDS 1..840

/gene="DIO2"

/note="It catalyzes the conversion of prohormone thyroxine

(3,5,3',5'-tetraiodothyronine, T4) to the bioactive

thyroid hormone (3,5,3'-triiodothyronine, T3) by outer

ring 5'-deiodination."

/codon_start=1

/product="iodothyronine deiodinase 2 (DIO2)"

/translation="MGLLSVDLLITVQVLPVFFSNCLFLALYDSVILLKHMVLFLSRS

KSGRGEWRRMLTLEGLRCVWNSFLLDAYKQVKLGGEAPNSSVIHIAKGSDGSSGSWKN

VGGKCGTKCHLLDFANSERPLVVNFGSATUPPFTSQLSAFSKLVEDFSGVADFLLVYI

DEAHPSDGWAAPGISPSSFEVKKHKSQEERCAAAHQLLEHFSLPPQCQVVADCMDNNA

NVAYGVSFERVCIVQRQKIAYLGGKGPFFYNLQEVRLWLEQNFSKRUNPFSTGVTSID

VSL"

BASE COUNT 191 a 209 c 227 g 213 t

ORIGIN

1 atgggtttgc taagtgtgga tttgttgatc acggttcagg tcttgccggt ctttttctcc

61 aattgcctct ttctcgcgct ctatgactct gtgattctgc tgaagcacat ggttctgttt

121 ctgagccgct ccaagtctgg gcgcggtgag tggcggagga tgctgaccct ggaggggctg

181 cgctgcgtct ggaacagctt cctcctggac gcctacaagc aggtcaaact gggaggagaa

241 gccccaaact ccagtgtaat ccacatagcc aaggggagtg atggcagtag tggcagctgg

301 aagaatgttg gtggaaagtg tggaaccaaa tgccaccttc tggattttgc caactcagag

361 cggccactgg tggtcaactt tggctcagct acctgaccac cgttcacaag ccagctgtca

421 gccttcagca agctggtgga ggacttctct ggtgtggctg actttctgtt ggtctacatc

481 gatgaagctc acccatcaga tggctgggct gcgcctggaa tctctccctc ttcattcgaa

541 gttaagaaac acaaaagcca ggaagaaaga tgtgcagctg ctcaccagct cctagagcac

601 ttttccttgc cgcctcagtg ccaagtggtg gctgactgca tggacaacaa tgcaaatgtg

661 gcctacgggg tttcatttga gcgagtatgc attgtgcaga gacaaaaaat tgcctacctg

721 gggggaaaag gcccctttt ctacaacctt caagaggtcc ggctttggct ggaacaaaac

781 ttcagcaaaa gatgaaatcc tttctccaca ggagttacgt caatcgatgt gtccctttaa

//

LOCUS Seq1 825 bp mRNA linear VRT 11-NOV-2019

DEFINITION iodothyronine deiodinase (DIO3) mRNA, complete cds.

ACCESSION Seq1

VERSION

KEYWORDS .

SOURCE Passer montanus (Eurasian tree sparrow)

ORGANISM Passer montanus

Eukaryota; Metazoa; Chordata; Craniata; Vertebrata; Euteleostomi;

Archelosauria; Archosauria; Dinosauria; Saurischia; Theropoda;

Coelurosauria; Aves; Neognathae; Passeriformes; Passeroidea;

Passeridae; Passer.

REFERENCE 1 (bases 1 to 825)

AUTHORS hao,y.

TITLE Direct Submission

JOURNAL Submitted (11-NOV-2019) Key Laboratory of Animal Physiology,

Biochemistry and Molecular Biology of Hebei Province, College of

Life Sciences, Hebei Normal University, Shijiazhuang 050024,

China., universities, No. 20, South Second Ring Road, Shijiazhuang,

Hebei Province, shijiazhuang, hebei province 050024, China

COMMENT Bankit Comment: ALT EMAIL:haoyinchao001@126.com

Bankit Comment: TOTAL # OF SEQS:1

##Assembly-Data-START##

Sequencing Technology :: Sanger dideoxy sequencing

##Assembly-Data-END##

FEATURES Location/Qualifiers

source 1..825

/organism="Passer montanus"

/mol_type="mRNA"

/db_xref="taxon:9160"

/country="China: Shijiazhuang,Hebei,province"

/altitude="176 m."

/collected_by="Yinchao Hao"

gene 1..825

/gene="DIO3"

/note="iodothyronine deiodinase 3 (DIO3)"

CDS 1..825

/gene="DIO3"

/note="It catalyzes the inactivation of thyroid hormone by inner ring deiodination of the prohormone thyroxine (T4) and the bioactive hormone 3,3',5-triiodothyronine (T3) to inactive metabolites, 3,3',5'-triiodothyronine (RT3) and 3,3'-diiodothyronine (T2), respectively."

/codon_start=1

/product="iodothyronine deiodinase 3 (DIO3)"

/translation="MLHSVGVHTLQLLTQAAACILLFPRFLLTAVMLWLLDFLCIRKK

MLTMPTADEAASASEEPPPDDPPVCVSDSNRMFTLESLKAVWHGQKLDFFKSAHVGSL

APNPEVIQLDGQKRLRILDFARGKRPLILNFGSCTUPPFMARLRSFQRLAAHFVDIAD

FLLVYIEEAHPSDGWVSSDAAYNIPKHQCLQDRLRAAQLMREGAPDCPLAVDTMDNAS

SAAYGACFERLYIIQEEKVMYQGGRGPEGYKISELRSWLDQYKTRLQSPSTVVIQV"

BASE COUNT 157 a 279 c 243 g 146 t

ORIGIN

1 atgctccact ccgtcggcgt tcacaccttg cagctgctca cccaggcggc cgcctgcatc

61 ctcctgtttc cccgcttcct gctcaccgcc gtgatgctct ggctcctgga ttttctgtgc

121 attaggaaga agatgctgac gatgcccacg gcggacgagg cggccagcgc cagcgaggag

181 ccgccccccg acgacccccc ggtctgcgtg tccgactcca accgcatgtt cacgctggag

241 tcgctgaaag ccgtgtggca cgggcagaag ctggacttct tcaagtcggc gcacgtggga

301 tccttggccc ctaaccccga ggtgatccag ctggacgggc agaagaggct ccgcatcctg

361 gacttcgccc gcggcaagag acccctcatc ctcaacttcg gcagctgcac ctgacccccg

421 ttcatggccc gcctgaggtc cttccagcgc ctggccgcgc acttcgtgga cattgccgac

481 ttcctgctgg tgtacattga agaagcacac ccgtccgacg gctgggtcag ctcggatgca

541 gcctacaaca tccccaagca ccagtgcctc caggacaggc tgcgggcagc tcagctgatg

601 agggaagggg cgcccgattg ccccctggcc gtggacacca tggacaatgc ttccagtgcc

661 gcctacggtg cctgcttcga gaggctctac atcattcagg aggagaaggt gatgtaccag

721 ggaggcagag gaccagaggg ctacaagatc tcggagctga ggagctggct agaccagtac

781 aaaacccggc tccagagccc cagcacggtg gtcatccaag tgtaa

//

LOCUS Seq1 272 bp mRNA linear VRT 31-MAR-2020

DEFINITION gonadotropin-releasing hormone I (GnRH‐I) mRNA, complete cds.

ACCESSION Seq1

VERSION

KEYWORDS .

SOURCE Passer montanus (Eurasian tree sparrow)

ORGANISM Passer montanus

Eukaryota; Metazoa; Chordata; Craniata; Vertebrata; Euteleostomi;

Archelosauria; Archosauria; Dinosauria; Saurischia; Theropoda;

Coelurosauria; Aves; Neognathae; Passeriformes; Passeroidea;

Passeridae; Passer.

REFERENCE 1 (bases 1 to 272)

AUTHORS hao,c.

TITLE Direct Submission

JOURNAL Submitted (31-MAR-2020) Key Laboratory of Animal Physiology,

Biochemistry and Molecular Biology of Hebei Province, College of

Life Sciences, Hebei Normal University, Shijiazhuang 050024,

China., universities, No. 20, South Second Ring Road, Shijiazhuang,

Hebei Province, shijiazhuang, hebei province 050024, China

COMMENT Bankit Comment: ALT EMAIL:haoyinchao001@126.com

Bankit Comment: TOTAL # OF SEQS:1

##Assembly-Data-START##

Sequencing Technology :: Sanger dideoxy sequencing

##Assembly-Data-END##

FEATURES Location/Qualifiers

source 1..272

/organism="Passer montanus"

/mol_type="mRNA"

/db_xref="taxon:9160"

/country="China: Shijiazhuang,Hebei,province"

/altitude="176 m."

/collected_by="Chenyang Hao"

gene <1..>272

/gene="GnRH1"

CDS <1..>272

/gene="GnRH1"

/note="It is a member of the gonadotropin-releasing

hormone (GnRH) family of peptides"

/codon_start=1

/product="gonadotropin-releasing hormone I (GnRH-I)"

/translation="MEKPRRMVATVLLCVLAVGLCLAQHWSFGLQPGGKRSAQVLLGP

FQVIPNEMEKLKEVQQSECPGSQQNPRIRDLKEAMERLAEEQGRRK"

BASE COUNT 67 a 64 c 100 g 41 t

ORIGIN

1 atggagaagc cccggaggat ggtggccact gtcctgctgt gtgtgctggc cgtggggctg

61 tgcctggccc agcactggtc cttcgggctc cagccagggg gcaagaggag cgcccaggtc

121 ctgctgggac cgttccaggt gattccaaat gaaatggaaa aattaaagga ggtgcagcag

181 agtgagtgcc caggctcgca gcagaacccc aggatcaggg atctgaagga agccatggag

241 aggctggcgg aggaacaagg cagaagaaag aa

//

LOCUS Seq1 522 bp mRNA linear VRT 31-MAR-2020

DEFINITION gonadotropin-inhibitory hormone pound (GnIH) mRNA, complete cds.

ACCESSION Seq1

VERSION

KEYWORDS .

SOURCE Passer montanus (Eurasian tree sparrow)

ORGANISM Passer montanus

Eukaryota; Metazoa; Chordata; Craniata; Vertebrata; Euteleostomi;

Archelosauria; Archosauria; Dinosauria; Saurischia; Theropoda;

Coelurosauria; Aves; Neognathae; Passeriformes; Passeroidea;

Passeridae; Passer.

REFERENCE 1 (bases 1 to 522)

AUTHORS Li,Y.

TITLE Direct Submission

JOURNAL Submitted (31-MAR-2020) Key Laboratory of Animal Physiology,

Biochemistry and Molecular Biology of Hebei Province, College of

Life Sciences, Hebei Normal University, Shijiazhuang 050024,

China., universities, No. 20, South Second Ring Road, Shijiazhuang,

Hebei Province, shijiazhuang, hebei province 050024, China

COMMENT Bankit Comment: ALT EMAIL:haoyinchao001@126.com

Bankit Comment: TOTAL # OF SEQS:1

##Assembly-Data-START##

Sequencing Technology :: Sanger dideoxy sequencing

##Assembly-Data-END##

FEATURES Location/Qualifiers

source 1..522

/organism="Passer montanus"

/mol_type="mRNA"

/db_xref="taxon:9160"

/country="China: shijiazhuang,Hebei,province"

/altitude="176 m."

/collected_by="Yaqing Li"

gene <1..>522

/gene="GnIH"

CDS <1..>522

/gene="GnIH"

/note="GnIH acts on hypothalamus GnRH neurons and

pituitary gonadotropins to inhibit gonadotropin synthesis

and release, thereby regulating bird reproduction"

/codon_start=1

/product="gonadotropin-inhibitory hormone (GnIH)"

/translation="MKVILSKKFILFALATVFFLTSNSMCLNEPMKSRLQSREDNDDK

YYEIKDNILEEKQRSLNFEDMEDWGTKDIIKMNHFTASKMPNSVANLPLRFGRNYPEE

RSIKPFSNLPLRFGRAFGENILNHAPKVSHRLGRSPLVKGSSQSLLNLPQRFGKSLAV

NLPRDIEESEPGI"

BASE COUNT 182 a 91 c 105 g 144 t

ORIGIN

1 atgaaagtca ttttatccaa gaagtttatt ctgtttgctt tagctacagt tttctttctc

61 acatcaaaca gcatgtgcct aaatgaacca atgaagtcca ggctgcagag cagagaagac

121 aatgatgata aatattatga gattaaagat aatattttgg aagaaaagca gaggagcctc

181 aattttgaag acatggaaga ctggggaaca aaagacatca ttaaaatgaa ccattttaca

241 gcaagcaaga tgccaaattc agttgctaat ttacctctta gatttggaag aaattatcca

301 gaagaaagaa gcattaaacc attttctaat ttgcccctga gatttggaag agcttttgga

361 gagaacatac ttaatcatgc tccaaaggta tcacacaggc ttgggagatc tccacttgtt

421 aaaggttcca gtcaatcact tctaaatttg ccacagagat ttgggaagtc actggctgtc

481 aatctgcctc gagacattga ggaatctgaa ccagggatat ga

//
